# Loss of breast cancer metastasis suppressor 1 promotes ovarian cancer cell metastasis by increasing chemokine receptor 4 expression

**DOI:** 10.3892/or.2011.1596

**Published:** 2011-12-19

**Authors:** XIU-JIE SHENG, YING-QUN ZHOU, QING-YUAN SONG, DONG-MEI ZHOU, QI-CAI LIU

**Affiliations:** 1 Department of Obstetrics and Gynecology, The Third Affiliated Hospital of Guangzhou Medical College, Guangzhou 510150; 2 Experimental Medical Research Center of Guangzhou Medical College, Guangzhou 510182, P.R. China

**Keywords:** ovarian neoplasm, breast cancer metastasis suppressor 1, RNA interference, metastasis, chemokine receptor 4

## Abstract

Breast cancer metastasis suppressor 1 (BRMS1) is a predominantly nuclear protein that differentially regulates the expression of multiple genes, leading to suppression of metastasis without affecting orthotopic tumor growth. It has been demonstrated that BRMS1 may be correlated with advanced ovarian cancer. The aim of this study was to investigate the mechanisms of BRMS1 involvement in ovarian cancer metastasis. We constructed a plasmid containing a short hairpin RNA (shRNA) against BRMS1 and transfected it into the ovarian cancer cell line OVCAR3. Real-time reverse transcription polymerase chain reaction (real-time PCR) and Western blot analyses demonstrated that BRMS1 expression was efficiently downregulated. Stable suppression of BRMS1 significantly enhanced cell adhesion, migration, invasion and angiogenesis. We also found that chemokine receptor 4 (CXCR4) was upregulated at both the mRNA and protein levels. When approaching for the mechanism, we discovered that activation of the nuclear factor-κB (NF-κB) signaling pathway mediated CXCR4 upregulation, as demonstrated by the electrophoretic mobility shift assay (EMSA). Collectively, these results suggest that attenuation of BRMS1 may play a critical role in promoting migration, invasion and angiogenesis of ovarian cancer cells and BRMS1 may regulate the metastatic potential at least in part through upregulation of CXCR4 via NF-κB activation. Restoration of BRMS1 function is thus a potential new strategy for treating human ovarian cancer.

## Introduction

Ovarian carcinoma is the leading cause of death from gynecological malignancies. The majority of ovarian cancer patient deaths are due to the irreversible physiological effects of metastases on normal organ function rather than from the primary tumor (1). Despite recently improved chemotherapeutic agents and an increased 5-year survival rate, ovarian cancer mortality remains unchanged. A better understanding of the metastatic mechanisms of ovarian cancer is therefore needed to determine effective therapeutic interventions to either eradicate or slow metastatic outgrowth.

Many factors are involved in regulating metastasis through diverse mechanisms. Among metastasis suppressors, breast cancer metastasis suppressor 1 (BRMS1) was originally shown to functionally suppress the metastatic capacities of breast cancer cells (2). Further studies showed that BRMS1 is not only a metastasis suppressor gene in breast cancer models but also in various other cancers, such as melanoma and ovarian cancer (3,4). Zhang *et al* (4) demonstrated that low levels of BRMS1 expression correlated with poor prognosis in ovarian cancer patients. They further showed that transfection of BRMS1 complementary DNA (cDNA) into the highly malignant ovarian carcinoma cell line HO-8910PM significantly reduced cell adhesion, motility and invasion *in vitro* and also decreased the incidence of lung metastasis without affecting tumor growth. BRMS1 is thought to regulate metastasis through multiple mechanisms, including restoration of gap junctions, reduction of phosphoinositide signaling, interaction with the histone deacetylase complex and regulation of the nuclear factor-κB (NF-κB) pathway (5–7). In particular, several metastasis-related genes were reported to be downregulated by BRMS1 through modulating the activity of NF-κB, including osteopontin (OPN), urokinase-type plasminogen activator (uPA), microRNA-146, interleukin-6 (IL-6) and chemokine receptor 4 (CXCR4) (8–12).

Chemokines are small cytokines that are characterized by their capacity to induce directional cellular migration towards a gradient of chemokines by binding to chemokine receptors. One of the most extensively studied chemokine receptors is CXCR4, which selectively binds the chemokine stromal cell-derived factor-1 (SDF-1) also known as CXCL12 (13). Recent evidence suggests that the SDF-1/CXCR4 pathway is involved in local invasion and metastasis of many cancers, including breast cancer, gastric cancer and ovarian cancer (14–16). Not only that, CXCR4 has been observed to promote angiogenesis by stimulating the secretion of several angiogenic factors, such as vascular endothelial growth factor and IL-6 (17,18). Interestingly, a recent study by Yang *et al* demonstrated that BRMS1 reduces CXCR4 expression in lung cancer cells via abrogation of NF-κB activation (12); however, the functional implications of BRMS1 and its relationship to the CXCR4 signaling pathway in ovarian neoplasms are not clear.

Therefore, we investigated the potential mechanisms of BRMS1-mediated metastasis suppression in ovarian cancer. In this study, recombinant plasmid containing short-hairpin RNA (shRNA) sequences targeting BRMS1 mRNA transcription regions was constructed and transfected into ovarian cancer cells. Their influences on cell adhesion, migration, invasion and angiogenesis were observed, and the expression of CXCR4 was detected. Finally, we employed an electrophoretic mobility shift assay (EMSA) to explore whether BRMS1 regulates CXCR4 expression through the NF-κB pathway. Our data indicate that BRMS1 negatively regulates metastatic potential at least in part through the suppression of NF-κB-dependent CXCR4 expression.

## Materials and methods

### Cell lines and cell culture

The human ovarian cancer cell line OVCAR3 (ATCC, USA) was grown in Dulbecco’s modified Eagle’s medium (DMEM) (Gibco, Invitrogen, USA) supplemented with 10% fetal bovine serum (FBS) (Gibco, Invitrogen) and antibiotics (100 U/ml penicillin and 100 μg/ml streptomycin). Human umbilical venous endothelial cells (HUVECs) were obtained from the Institute of Biochemistry and Cell Biology of the Chinese Academy of Science (Shanghai) and cultured in Kaighn’s modified Ham’s F-12K medium (Mediatech, Manassas, VA, USA) supplemented with endothelial cell growth supplement (BD Biosciences, Canada) and 10% FBS. Cultures were tested and shown free of mycoplasma contamination. All cells were maintained in 5% CO_2_ atmosphere at 37°C. For all functional and biological assays, cells with >95% viability were used at 70–90% confluence.

### Plasmids construction

Based on the preliminary results of screening out effective silencing siRNA sequences, the following double-stranded RNA oligonucleotides specific for the BRMS1 coding region were used: 5′-CACCGTTCGTACTT ATTCCTGATCACATCCTTCAAGAGAGGATGTGATCAG GAATAAGTACGAATTTTTTG-3′ (sense), 5′-GATCCAA AAAATTCGTACTTATTCCTGATCACATCCTCTCTTG AAGGATGTGATCAGGAATAAGTACGAAC-3′ (antisense); Negative control sequences with no significant homology to the BRMS1 gene and which had the sequence not present in the human, mouse or rat genome databases were: 5′-CACCGT TCTCCGAACGTGTCACGTCAAGAGATTACGTGACACG TTCGGAGAATTTTTTG-3′ (sense), 5′-GATCCAAAAAAT TCTCCGAACGTGTCACGTAATCTCTTGACGTGACACG TTCGGAGAAC-3′ (antisense). All DNA chains were synthesized by GenePharma Co. (Shanghai, China). The plasmids were extracted and the accuracy of the constructs was confirmed by DNA sequencing.

### Cell transfection

According to the manufacturer’s protocol for Lipofectamine 2000 (Invitrogen), pGPU6/GFP/Neo-BRMS1 or pGPU6/GFP/Neo-NC were transfected into OVCAR3 cells. After 6 h, the cultures were replaced with 2 ml fresh medium supplemented with 10% FBS and antibiotics. Then the cells were visualized under fluorescence microscopy. After 48 h, 600 μg/ml G418 (Sigma, USA) was added to the medium for selecting stable transfectants, and individual clones were isolated and maintained in a medium containing 300 μg/ml G418. Real-time PCR and Western blotting were applied to analyze BRMS1 mRNA and protein levels, respectively. The stably transfected OVCAR3 cells were named BRMS1-shRNA (transfected with pGPU6/GFP/Neo-BRM-S1) and NC-shRNA, respectively.

### Real-time reverse transcription polymerase chain reaction (real-time PCR)

Total RNA from cells was extracted with TRIzol Reagent (Invitrogen) following the manufacturer’s instruction. cDNA was synthesized from total RNA using the PrimeScript RT reagent kit (Takara, Japan). The cDNA specimens were amplified using the SYBR Premix Ex Taq™ (Takara). GAPDH gene was used as an internal control for standardization in triplicate. Cycle conditions were: 95°C for 30 sec, followed by 40 cycles of 95°C for 5 sec, 60°C for 34 sec and finally 95°C for 15 sec, 60°C for 1 min. PCR amplification was performed on the ABI 7500 Sequence Detection System (PE Applied Biosystems, Foster City, CA, USA). The comparative Ct (ΔΔCT) method was used to determine the expression fold change. The sequences of the primers used were as follows: BRMS1 forward: 5′-ATGCCTGTCCAGCCTCC AAG-3′ and reverse 5′-GCGTCGCTCATAGTCCTCATCA-3′; CXCR4 forward: 5′-GGTGGTCTATGTTGGCGTCT-3′ and reverse 5′-CTCAGTGGAAACAGATGAAT-3′; GAPDH forward: 5′-GCACCGTCAAGGCTGAGAAC-3′ and reverse 5′-TGGT GAAGACGCCAGTGGA-3′.

### Western blot analysis

Total cellular proteins from the cells were obtained using RIPA lysis buffer (Santa Cruz Biotechnology, USA) containing a cocktail of proteinase inhibitors and phosphatase inhibitors. Protein concentrations were measured using the BCA protein assay (Sigma). The proteins were subjected to 10% SDS denatured polyacrylamide gel and transferred onto PVDF membranes. Membranes were blocked in 5% non-fat milk for 1 h at 4°C and blotted with rabbit anti-human antibody at the recommended dilution BRMS1 (1:500, BioWorld, USA), CXCR4 (1:100, Epitomics, USA) and β-actin (1:500, BioWorld), and subsequently incubated with the appropriate secondary antibody. After washing with TBST, visualization of the second antibody was performed using a chemiluminescence detection procedure according to the manufacturer’s protocol (Amersham Biosciences, Japan). The LabWorks™ Image Acquisition and Analysis Software (UVP, USA) was used to quantify band intensities. β-actin was used as a loading control.

### Cell adhesion assay

For this assay, 96-well plates were incubated with 50 μl (30 μg/ml) BD Matrigel™ Matrix (BD Biosciences, Germany) at 4°C overnight, then washed with PBS twice and blocked with 1% BSA for 1 h at 37°C. Cells were trypsinized and seeded at 1×10^5^/ml to each coated well and incubated for 2 h in 5% CO_2_ atmosphere at 37°C, then rinsed three times with PBS to remove non-adherent cells. Each well with 100 μl medium was added 20 μl 3-(4,5-dimethylthiazol-2-yl)-2,5-diphenyltetrazolium bromide (MTT), further incubated for 4 h, then the MTT was removed and 150 μl dimethylsulfoxide (DMSO) was pipetted into each well. The optical density (OD) was measured at 570 nm with a microplate reader. The OD values were propotional to the number of cells with adhesion; five duplicate wells were set up for each group.

### Migration assay

Cell migration was assessed by adding 5×10^4^ cells into the upper chamber of an 8-μm pore size Transwell insert (Corning, USA) in serum-free media. These inserts were placed in wells with serum-containing media. After seeding for 24 h, non-migrating cells were removed from the upper surface of the filter with a cotton-tipped swab. The cells on the lower surface of the filter were fixed in 4% paraformaldehyde and stained using crystal violet staining solution. Five random fields were counted at ×100 magnification. All the data presented are from at least three independent experiments performed in duplicate.

### Matrigel invasion assay

Transwell inserts (8-μm pore size) coated with 30 μl Matrigel were placed in wells as previously described. In the top chamber, 5×10^4^ cells were plated in serum-free media and incubated with serum-containing media as a chemoattractant in the bottom chamber. Cells were then incubated at 37°C and allowed to invade through the Matrigel barrier for 24 h. After incubation, non-invading cells were removed using a cotton swab. Filters were fixed and stained with crystal violet staining solution, and five random fields were counted at ×100 magnification. All the data presented are from at least three independent experiments performed in duplicate.

### In vitro tube formation assay

OVCAR3 cells were cultured in 6-well plates with fresh complete medium for 24 h and 1 ml conditioned medium was collected. For tube formation assay, the 48-well plates were coated with Matrigel (100 μl per well) and kept in 5% CO_2_ atmosphere at 37°C for 30 min. Then, 5×10^4^ HUVECs were suspended in 500 μl conditioned medium and applied to the pre-coated 48-well plates. After incubation at 37°C for another 24 h, images were captured under a microscope and the tubular structures formed in the Matrigel were counted at ×100 magnification in five random fields.

### Electrophoretic mobility shift assay (EMSA)

The assay is based on that DNA-protein complexes migrate slower than unbound DNA double-stranded oligonucleotides on a native polyacrylamide gel, resulting in a ‘shift’ in the migration of the labeled DNA band. The detection of bands was performed by the LightShift™ Chemiluminescent EMSA kit (Pierce, USA) that used a non-isotopic method to detect DNA-protein interactions. Nuclear extracts were prepared from OVCAR3 cells knockdown of BRMS1 and the control sample. Nuclear proteins were incubated at room temperature for 10 min with oligonucleotide probe bearing an NF-κB binding sequence on the CXCR4 promoter (5′-TCCCCTGGGCTTCCCAAGCC-3′). The probe was labeled with a biotin at its 5′-end. Another oligonucleotide with the same sequence but without labeling was used as a competitive sequence at 500-fold concentration. After the reaction the DNA-protein complexes were subjected to a 6.5% native polyacrylamide gel electrophoresis and transferred to a nylon membrane. Then the membrane was immediately cross-linked for 15 min on a UV transilluminator equipped with 312 nm bulbs. Finally, a chemiluminescent detection method utilizing a luminal/enhancer solution and a stable peroxide solution was used as described by the manufacturer and membranes were exposed to X-ray films for 2–5 min before developing.

### Statistical analysis

All experiments were performed at least in triplicate and data were compiled from three separate experiments. The results were calculated as means ± SD. All statistical analyses were determined by one-way ANOVA using the SPSS16.0 software. A P-value <0.05 was considered significant.

## Results

### Specific inhibition of BRMS1 expression by BRMS1-shRNA

**Figure 1:**
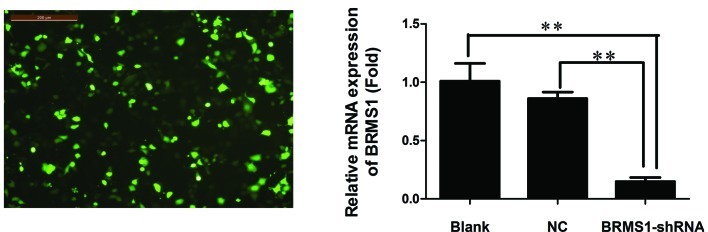

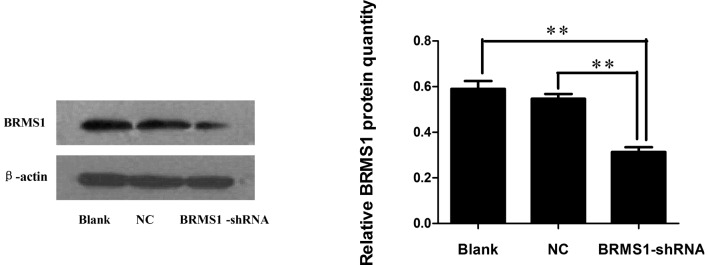
Suppression of BRMS1 expression by small hairpin RNA (shRNA) in OVCAR3 cells. (A) Representative pictures of OVCAR3 cells 24 h after transfection with BRMS1-shRNA (x100 magnification). (B) The relative expression of BRMS1 mRNA was analyzed by real-time PCR. (C) Total protein was extracted and measured by Western blot analysis. β-actin was used as a loading control. Both an untransfected control (blank control) and a non-specific sequence control (NC) were also included. Data shown are the means ± SD of a representative experiment performed in triplicate (^**^P<0.01).

Plamid vectors expressing either BRMS1-shRNA or non-specific sequence control (NC) shRNA were constructed and transfected into OVCAR3 cells. After 24 h, high transfection efficiency was observed by fluorescence microscopy (Fig. 1A). To determine silencing efficiency, the expression levels of BRMS1 mRNA and protein were measured by real-time PCR and Western blot analysis, respectively. The BRMS1 mRNA level declined significantly in the BRMS1-shRNA transfected cells, with an average inhibition of 85.15% compared to the blank control group (P<0.01, Fig. 1B). BRMS1 protein expression was also decreased, with an average inhibition of 46.67% in the BRMS1-shRNA group (P<0.01, Fig. 1C). These results suggested that pGPU6/GFP/Neo-BRMS1 could effectively suppress BRMS1 expression at both the mRNA and protein levels in OVCAR3 cells.

### Effect of BRMS1-shRNA on adhesion

**Figure 2:**
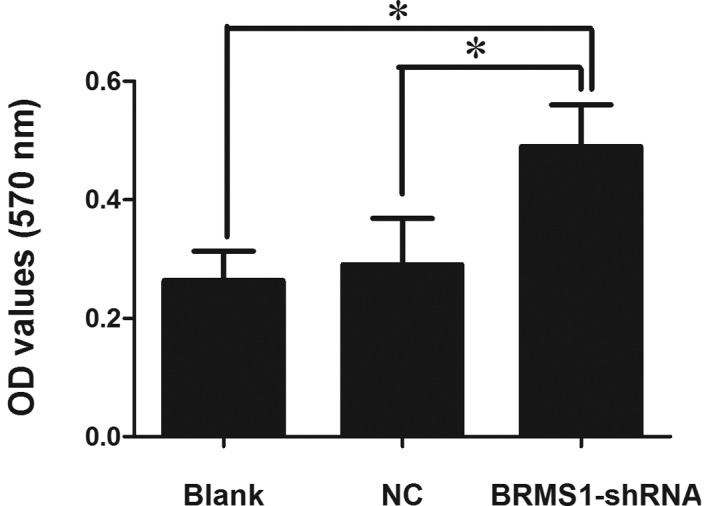
Knockdown of BRMS1 expression enhances the adhesion of OVCAR3 cells. Adhesion was examined by determining the optical densities (OD) at 570 nm. OVCAR3 cells stably expressing BRMS1-shRNA exhibited a significant increase in adhesion. Results are expressed as the average optical densities (means ± SD, ^*^P<0.05).

Cancer cell adhesion to the subendothelial extracellular matrix is an important step in metastasis formation. To assess the potential involvement of downregulation of BRMS1 expression on adhesion, a cell adhesion assay was employed. Our results showed that BRMS1-shRNA cells markedly enhanced cell adhesion to the Matrigel matrix. The OD values at 570 nm were proportionate to the number of attached cells. Cell adhesion of BRMS1-shRNA transfected OVCAR3 cells was increased by 1.88-fold compared to the blank control cells (Fig. 2).

### Effect of BRMS1-shRNA on migration and invasion

**Figure 3:**
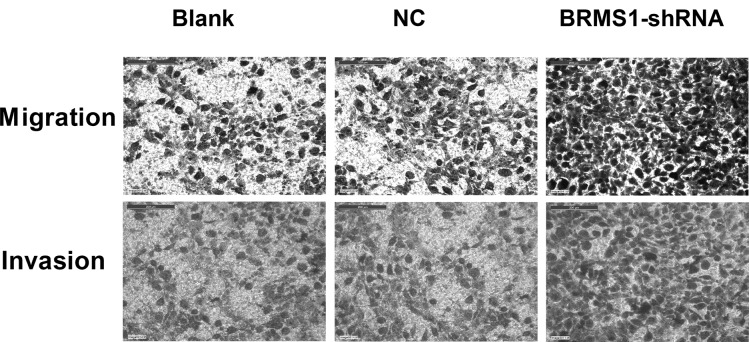

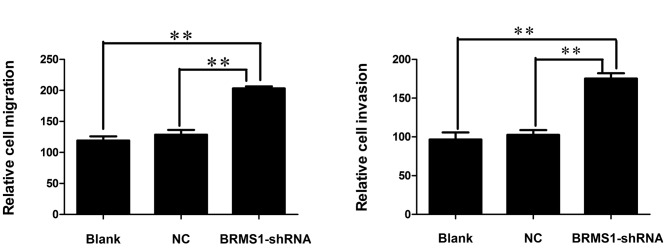
Inhibition of BRMS1 expression promotes migration and invasion of OVCAR3 cells. (A) Representative crystal violet staining of migrated and invaded cells. (B) The average migrated and invaded cell numbers of OVCAR3 cells transfected with BRMS1-shRNA were significantly higher than those of both the negative and blank control groups (migration: 203.33±5.68, 128.67±13.43 and 119.33±11.59; invasion: 175.33±11.72, 102.67±10.41 and 96.67±15.63). Data are represented as the means ± SD of three independent experiments (^**^P<0.01).

Migration and Matrigel invasion assay were performed to examine the impact of BRMS1-shRNA on cell migration and invasion, respectively. As shown in Fig. 3, BRMS1 knockdown increased OVCAR3 cell migration by 1.7-fold compared to untreated cells. The Matrigel invasion assay results demonstrated that the invasiveness of cells treated with BRMS1-shRNA increased by 1.81-fold compared to the control group. Taken together, these data indicated that BRMS1-shRNA promoted motility and invasion of OVCAR3 cells.

### Effect of BRMS1-shRNA on angiogenesis

**Figure 4:**
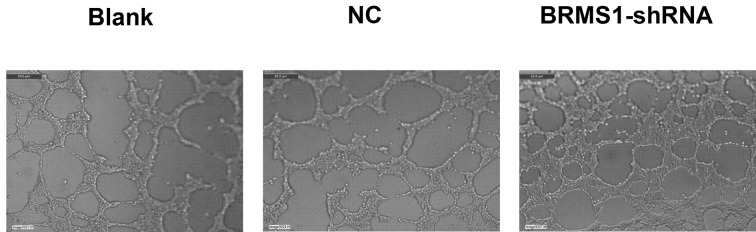

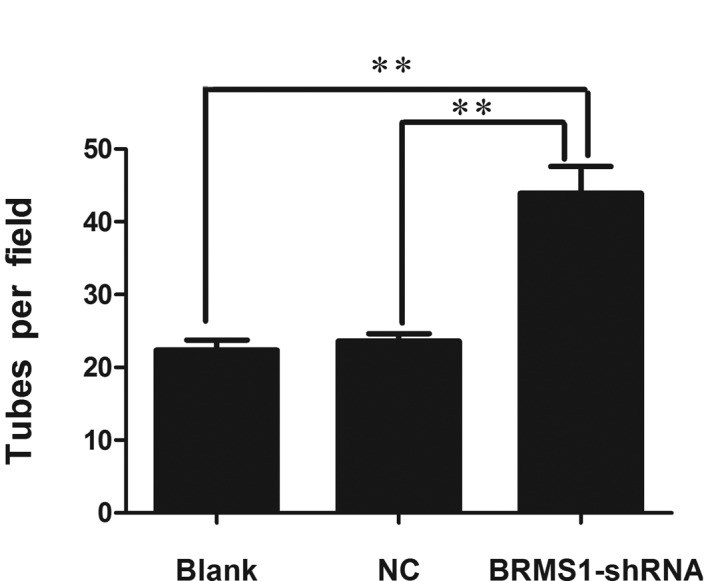
BRMS1 silencing induces ovarian cancer cells angiogenesis. (A) Capillary-like tubes were observed and photographed. BRMS1 knockdown in ovarian cancer cells promoted HUVECs tube formation. (B) The numbers of tubes formed per field were counted in 5 random fields for the BRMS1 knockdown, negative control and blank control groups (43.93±6.33, 23.60±1.78 and 22.33±2.52). Data are presented as means ± SD from three independent experiments (^**^P<0.01).

Angiogenesis plays a critical role in the growth and metastatic potential of all solid tumors. To determine the effects of BRMS1 silencing on ovarian cancer cell angiogenesis, we utilized the tube formation assay. Compared to the corresponding control, the average number of complete tubular structures formed by HUVECs was increased by 1.97-fold in conditioned medium in OVCAR3 cells transfected with BRMS1-shRNA. These data indicated that inhibition of BRMS1 significantly enhanced the angiogenic capacity of the ovarian cancer cells (Fig. 4).

### Upregulation of CXCR4 by BRMS1-shRNA

**Figure 5:**
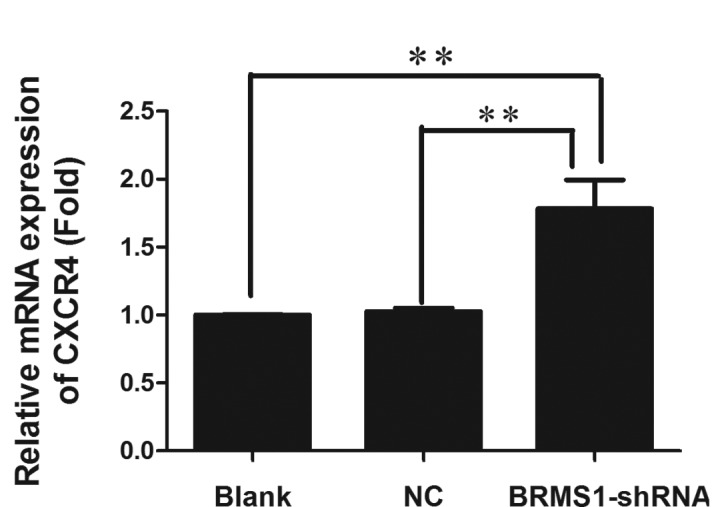

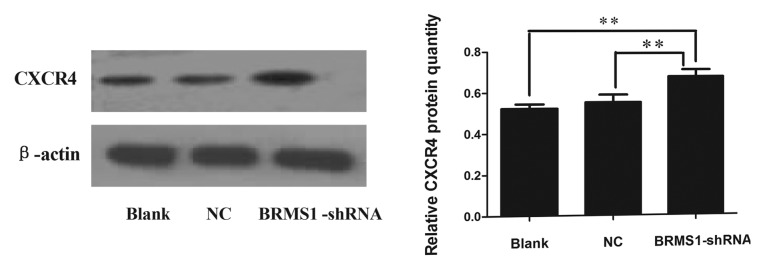
Suppression of BRMS1 upregulates CXCR4 at both the transcriptional and translational levels. (A) Total RNA was extracted from OVCAR3 cells transfected with either BRMS1-shRNA or NC-shRNA, and CXCR4 mRNA expression was measured by real-time RT-PCR. When BRMS1 was blocked, CXCR4 mRNA was increased compared to control cells (^**^P<0.01). (B) Protein was extracted from OVCAR3 cells transfected with either BRMS1-shRNA or NC-shRNA and CXCR4 protein level was measured by Western blot analysis. β-actin was used as a loading control. Results revealed that BRMS1 silencing in OVCAR3 cells elevated CXCR4 protein by 1.26-fold (^**^P<0.01).

Recently, BRMS1 was shown to regulate metastatic potential through the down-regulation of CXCR4 (12). To determine whether loss of BRMS1 regulated CXCR4 expression in ovarian cancer, CXCR4 mRNA and protein levels of OVCAR3 cells transfected with either BRMS1-shRNA or a negative control were measured by real-time RT-PCR and Western blot analysis, respectively. As shown in Fig. 5, CXCR4 mRNA was 1.78-fold higher in BRMS1-shRNA transfected cells compared to the blank control group. Furthermore, Western blot analysis revealed that BRMS1 silencing in OVCAR3 cells elevated CXCR4 protein levels by 1.26-fold. These results elucidated that knockdown of BRMS1 upregulated CXCR4 at both the transcriptional and translational levels. Taken together, our data indicate that loss of BRMS1 expression induces adhesion, migration, invasion and angiogenesis of OVCAR3 cells, which may be due to upregulation of CXCR4.

### BRMS1 knockdown promotes CXCR4 expression through activation of the NF-κB signaling pathway

**Figure 6:**
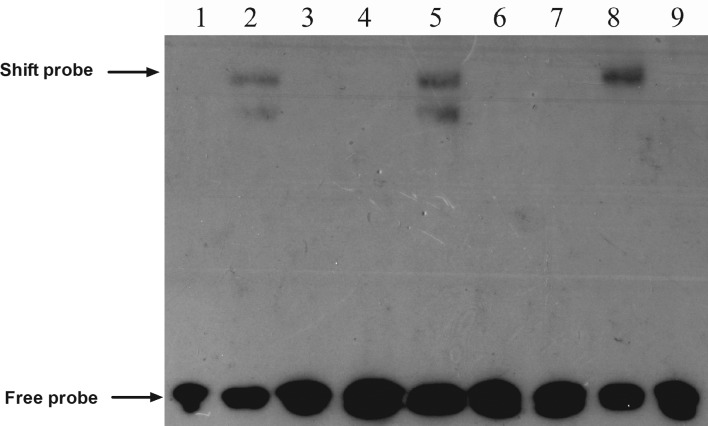
Inhibition of BRMS1 activates NF-κB activity. NF-κB binding to the CXCR4 promoter in nuclear extracts was measured by EMSA. BRMS1 knockdown activated NF-κB binding to the CXCR4 promoter, whereas a competitive sequence inhibited the DNA binding reaction. Lanes 1, 4 and 7, probe labeled with biotin; Lane 2, probe + nuclear extract from untreated OVCAR3 cells; Lane 3, probe + competitive probe + nuclear extract from untreated OVCAR3 cells; Lane 5, probe + nuclear extract from OVCAR3 cells transfected with a non-specific sequence; Lane 6, probe + competitive probe + nuclear extract from OVCAR3 cells transfected with a non-specific sequence; Lane 8, probe + nuclear extract from OVCAR3 cells transfected with BRMS1- shRNA; Lane 9, probe + competitive probe + nuclear extract from OVCAR3 cells transfected with BRMS1-shRNA.

A prior study revealed that NF-κB promoted breast cancer migration and invasion by directly upregulating CXCR4 expression (19). Therefore, we chose to perform an EMSA to explore whether BRMS1 regulates CXCR4 expression through the NF-κB pathway in ovarian cancer cells. The EMSA results suggested that BRMS1 knockdown increased the DNA binding activity of NF-κB to the CXCR4 promoter, compared to the control groups, whereas the unlabeled competitive sequence markedly inhibited this binding (Fig. 6).

## Discussion

Metastasis is a multistep process involving dissociation of malignant cells in the primary tumor, local invasion, angiogenesis, intravasation, survival in the circulation, extravasation and proliferation at a secondary site (20). These processes are modulated by many factors among which the metastasis suppressors are of particular importance for elucidating the underlying genetic and molecular biological mechanisms of metastasis.

The metastasis suppressor gene BRMS1 was discovered by Seraj *et al* (2) while studying the non-random amplifications and deletions in chromosome 11 using differential display. It is located on chromosome 11q 13.1–13.2 and consists of 10 exons and 9 introns spanning approximately 7 kb. Previous research showed that introducing BRMS1 into the highly metastatic ovarian cancer cell line HO-8910PM significantly suppressed adhesion, motility and local invasion without affecting tumor growth *in vitro* (4). Moreover, a recent report suggested that BRMS1 was associated with tumor angiogenesis. Loss of BRMS1 resulted in deficient suppression of vasculogenesis and contributed to melanoma metastasis (11). BRMS1 has also been shown to reduce the capacity of multiple human cancer cell lines to metastasize to the lymph nodes, lungs and/or bone in experimental models (2,21,22). In addition, BRMS1 has clinical relevance for some tumor types. BRMS1 mRNA expression was downregulated in breast tumor tissues (7) and in breast cancer brain metastases (23). Hicks *et al* (24) claimed that attenuation of BRMS1 expression in breast carcinomas was associated with reduced disease-free survival in the context of hormone receptor-negativity or HER2 overexpression. Furthermore, Zhao and Wang (25) observed that BRMS1 expression in ovarian serous adenocarcinoma was significantly lower than in both normal ovarian tissue and benign ovarian tumor tissue. BRMS1 was correlated with surgical stage, lymph node metastasis and tumor size. Another study determined that both BRMS1 mRNA and protein levels were diminished in non-small cell lung cancer (NSCLC) compared to the adjacent non-cancerous lung. Preservation of BRMS1 expression was accordingly associated with improved survival of NSCLC patients (26). Recently, an increasing number of studies have demonstrated the potential of using BRMS1 as a prognostic marker and therapeutic target for breast cancer (27), ovarian cancer (4), melanoma (11) and NSCLC (26). Together, the data provide compelling evidence that BRMS1 is an effective metastasis suppressor in tumors; however, the mechanistic basis for its metastasis-suppressive function in human ovarian cancer is poorly defined.

In this study, we employed RNA interference (RNAi) technology to knock down endogenous BRMS1 expression and analyzed the influence of BRMS1 on the metastatic behavior of ovarian cancer cells. Due to the stability and long-term effectiveness of shRNA, BRMS1-shRNA was constructed and transfected into the human ovarian cancer cell line OVCAR3. Our data revealed that the expression of BRMS1 mRNA and protein was decreased in OVCAR3 cells following BRMS1-shRNA transfection, with inhibition rates of 85.15% at the mRNA level and 46.67% at the protein level. We then focused on cell adhesion, migration, invasiveness and angiogenesis, all of which are essential steps for the establishment of metastasis. We found that BRMS1 silencing increased adhesion, migration and invasion, and induced vascularization of ovarian cancer cells. Consistent with results reported in the literature, we determined that BRMS1 is indeed an effective metastasis suppressor in ovarian cancer.

Numerous studies have confirmed that many BRMS1 downstream targets are involved in regulating tumor progression and metastatic behaviors. It has also been reported that these processes are associated with NF-κB signaling pathways. Cicek *et al* (7) demonstrated that BRMS1 expression led to the inhibition of IκBα phosphorylation and degradation and subsequently to a reduction in NF-κB nuclear translocation. Expression analysis has indicated that the OPN is decreased when BRMS1 is overexpressed in MDA-MB-435 cells; interestingly, a mechanism by which BRMS1 reduces OPN expression levels is via abrogation of NF-κB activation (8). Another study revealed that BRMS1 expression stimulated p65 dissociation from the NF-κB binding site of the uPA promoter, which resulted in reduced transactivation of uPA expression (9). Moreover, BRMS1 has been shown to negatively regulate melanoma angiogenesis by suppressing NF-κB activity and IL-6 expression (11). Perhaps most interestingly, BRMS1 was shown to reduce CXCR4 expression via abrogation of NF-κB signaling, which led to metastasis suppression in lung cancer cells (12). CXCR4 is a seven-domain transmembrane chemokine receptor that is predominantly expressed on lymphocytes where it activates chemotaxis. SDF-1 is the only physiological ligand for CXCR4. The SDF-1/CXCR4 axis has been recently shown to be involved in stimulating multiple metastatic processes in many different neoplasms, including migration, invasion, angiogenesis and proliferation (13–16). Chu *et al* (17) also demonstrated that CXCR4 overexpression increased vascularity, which may help promote human basal cell carcinoma metastasis. Conversely, both knockdown of CXCR4 and use of a neutralizing antibody against CXCR4 in ovarian carcinoma decreased invasion (28). Moreover, a prior report showed that NF-κB could promote migration and organ-specific homing of cancer cells through the induction of CXCR4. The NF-κB binding site has also been identified in the proximal region of the CXCR4 promoter and is postulated to play a role in CXCR4 expression in human breast cancer cells (19,28,29). Because both BRMS1 and CXCR4 are involved in regulating the NF-κB signaling pathway, we hypothesized that BRMS1 might modulate metastasis of ovarian cancer cells in part by regulating CXCR4 expression. Our data suggested that inhibiting BRMS1 in OVCAR3 cells could lead to the upregulation of CXCR4. We further investigated whether the increase in CXCR4 expression resulting from BRMS1 silencing was due to activation of the NF-κB pathway. To address this question we used an EMSA targeting NF-κB binding in the CXCR4 promoter. We determined that blocking BRMS1 obviously increased NF-κB binding to the CXCR4 promoter compared to the parental cells, whereas an unlabeled competitive sequence markedly inhibited this binding. Taken together, these data provided mechanistic support for our hypothesis that BRMS1 regulates CXCR4 expression through the NF-κB pathway.

In summary, we report that knockdown of BRMS1 in ovarian cancer cells is associated with upregulation of CXCR4 mediated by NF-κB activation, which then increases the metastatic potential. Our results contribute to the better understanding of the tumor-suppressive functions of BRMS1 in ovarian cancer and suggest that BRMS1 restoration may be a promising approach for anti-metastasis therapy for human ovarian cancer.
